# Fusion of Visible and Infrared Aerial Images from Uncalibrated Sensors Using Wavelet Decomposition and Deep Learning

**DOI:** 10.3390/s24248217

**Published:** 2024-12-23

**Authors:** Chandrakanth Vipparla, Timothy Krock, Koundinya Nouduri, Joshua Fraser, Hadi AliAkbarpour, Vasit Sagan, Jing-Ru C. Cheng, Palaniappan Kannappan

**Affiliations:** 1 Department of Electrical and Computer Engineering, University of Missouri, Columbia, MO 65211, USA; 2 Department of Computer Science, Saint Louis University, St. Louis, MO 63103, USA; 3 Department of Earth, Environmental and Geospatial Sciences, Saint Louis University, St. Louis, MO 63108, USA; 4 Engineer Research and Development Center, U.S. Army Corps of Engineers, Vicksburg, MS 39180, USA

**Keywords:** VIS-IR multi-modal fusion, image matching, spectral decomposition, deep neural network (DNN)

## Abstract

Multi-modal systems extract information about the environment using specialized sensors that are optimized based on the wavelength of the phenomenology and material interactions. To maximize the entropy, complementary systems operating in regions of non-overlapping wavelengths are optimal. VIS-IR (Visible-Infrared) systems have been at the forefront of multi-modal fusion research and are used extensively to represent information in all-day all-weather applications. Prior to image fusion, the image pairs have to be properly registered and mapped to a common resolution palette. However, due to differences in the device physics of image capture, information from VIS-IR sensors cannot be directly correlated, which is a major bottleneck for this area of research. In the absence of camera metadata, image registration is performed manually, which is not practical for large datasets. Most of the work published in this area assumes calibrated sensors and the availability of camera metadata providing registered image pairs, which limits the generalization capability of these systems. In this work, we propose a novel end-to-end pipeline termed *DeepFusion* for image registration and fusion. Firstly, we design a recursive crop and scale wavelet spectral decomposition (WSD) algorithm for automatically extracting the patch of visible data representing the thermal information. After data extraction, both the images are registered to a common resolution palette and forwarded to the DNN for image fusion. The fusion performance of the proposed pipeline is compared and quantified with state-of-the-art classical and DNN architectures for open-source and custom datasets demonstrating the efficacy of the pipeline. Furthermore, we also propose a novel keypoint-based metric for quantifying the quality of fused output.

## 1. Introduction

**Figure 1:**
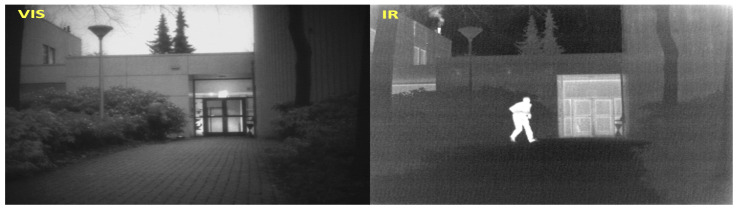
Image on the left shows the visible image from Kittler dataset where the person in the scene has low contrast against the background dark foliage due to low-lighting conditions. The same person is clearly visible in the corresponding thermal image on the right.

Electronic image sensors capture the ambient information over specific wavelength ranges for which they are optimized to operate. For example, visible (VIS), RGB, or color sensor systems capture information in spectral range of (400–700) nm wavelength, which is close to the functional range of the human retina, while infrared (IR) or thermal sensors operate between (1–14) μm wavelength. Visible sensors capture the light reflected by the object towards the camera aperture and the image is focused on the detector. The information obtained is directly proportional to the reflectivity of the target. Infrared systems on the other hand, capture thermal gradient information of the target. The quality of the image obtained is directly proportional to the efficiency of target emissivity. These two modalities capture information using different physical material properties and cannot be directly correlated, i.e., in a nighttime-captured image pair, a bright white-hot object in IR may be a low visibility person in VIS as shown in Figure 1. Fusing VIS and IR images can provide better scene understanding in challenging degraded environments and weather conditions. Fused images are further processed for several real-time applications such as object tracking [1,2,3,4] image segmentation [5,6,7], medical imaging [8,9,10], and agricultural applications [11,12]. Fusion requires the multi-modal image pairs to be registered accurately to reduce artifacts in the fused image. Image registration is one of the most challenging tasks in computer vision and still an open problem. Presently, image registration is performed manually using the camera metadata or visual cues. Most of the published literature on image fusion assumes the availability of registered VIS-IR image pairs which may not always be the case. This limits the generalization capabilities of the existing methods. In this work, we develop a novel automatic field-of-view (FOV) alignment method for image registration based on wavelet spectral decomposition (WSD) and post-processing followed by VIS-IR image fusion using a custom DNN architecture.

**Figure 2:**
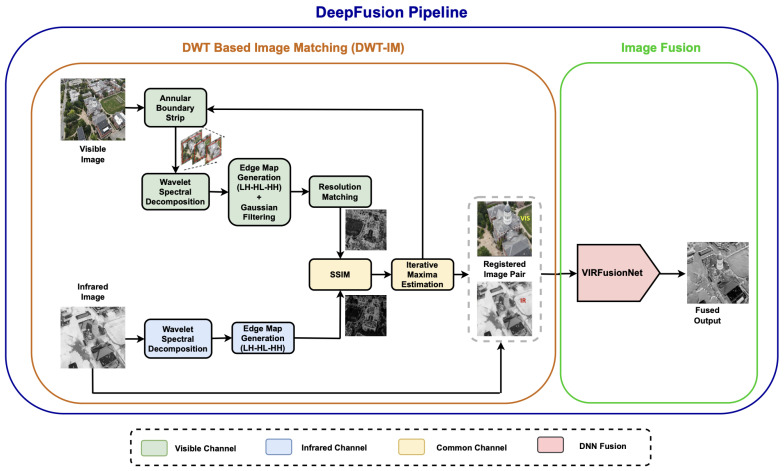
Architecture of the proposed DeepFusion pipeline. It consists of two modules: DWT-based image matching and VIS-IR Image Fusion. The high-resolution visible image is iteratively stripped and passed onto wavelet spectral decomposition module to decompose the image into spectral bands. The information from high-frequency bands is fused to generate a comprehensive edge map. The edge map in the visual channel is gaussian-filtered to smooth out internal edges. The processed image is scaled back to the infrared resolution. The SSIM index for the pre-processed infrared image and visible image are calculated iteratively, terminating the loop when the SSIM index crosses a local maxima. The stripped visible patch is processed according to the iteration number to generate the registered multi-modal image pair. The image pair is then passed into *VIRFusionNet* DNN to generate the fused output.

The relationship between registered image pairs can be assessed visually, but there is no mathematical framework yet that can characterize the underlying similarity numerically. A model relating the VIS and IR modalities was developed by Iwashitha et al. [13], under some assumptions. They proposed that both the reflected and emitted energy from the object are additive components of the total energy incident on the object of interest and given by,

(1) $$I_{Total\;Energy}=R_{VIS}+E_{IR}$$

where $I_{Total\;Energy}$ is the total energy incident on the object, $R_{VIS}$ is the reflected energy, and $E_{IR}$ is the absorbed and emitted energy. To assert the validity of the proposed relationship between the individual components, they made the following two assumptions: (a) the energy directed towards the object of interest has constant intensity, and (b) the refracted component of energy is zero, i.e., all the absorbed energy is emitted as infrared radiation. Although these conditions rarely prevail in the real-time environment, they empirically point to a possibility of data correlation between VIS-IR images. These two components (VIS, IR) of energy operate in a complementary way, with the visual system capturing maximum information about the object of interest during the day in terms of color, contrast, etc., and the infrared system capturing the information efficiently during nighttime in terms of temperature gradients. So, to design a system capable of all-day, all-weather operation, both visual and infrared systems have to be jointly deployed, and the information from both the sensors has to be judiciously fused to maximize entropy of the captured information. In this work, we propose an end-to-end image matching and fusion pipeline termed DeepFusion shown in Figure 2. The remainder of the paper is organized as follows: Section 2 presents related work published in this area. Section 3 focuses on a proposed algorithm for resolution mapping and image fusion. Section 4 discusses the experiments and results in detail followed by the conclusions in Section 5 and references.

## 2. Related Work

In this work, we propose an end-to-end image fusion pipeline with three components: multi-modal image matching, image fusion, and fusion performance analysis. We discuss related works in these areas for contextual relevance.

### 2.1. Multi-Modal Image Matching

Image processing literature can be broadly divided into classical methods, feature vector-based methods, and DNN-based methods for both single-mode and multi-mode applications. Multi-modal systems intrinsically have different modality variance and data representation. For example, in medical imaging, multi-modal matching might mean extracting and matching the information from CT and MRI scans [14]. Remote sensing and computer vision literature primarily deal with visible and infrared systems for surveillance and tracking [15] and hyper or multi-spectral systems for crop yield estimation [16,17], species distribution [18], etc. Classical methods work with pixel-level data to find a correlation between the multi-modal data. Feature vector-based methods try to represent the data as unique descriptors for distance-based correlation and DNN-based methods generate adaptable, high-dimensional feature vectors, which capture much more information than classical and feature-based methods from the input images for image matching.

#### 2.1.1. Classical Methods

Classical methods rely on transform algorithms, weighted averaging and correlation-based methods which directly work with pixel level information to find a correspondence between the two images. However, they are highly susceptible to variations in illumination conditions, scaling, and orientation. Ye et al. [19] proposed an FFT-based local feature representation in the frequency domain to calculate the similarity in the spectral domain. In [20] authors present a DCT-based descriptor matching for robust feature tracking in visible wide-area motion imagery. In general, a robust similarity measure plays a pivotal role in data-based matching algorithms. SSIM and normalized cross-correlation are examples of methods used in classical techniques for image comparison in the classical domain.

#### 2.1.2. Handcrafted Feature Vector-Based Methods

**Figure 3:**
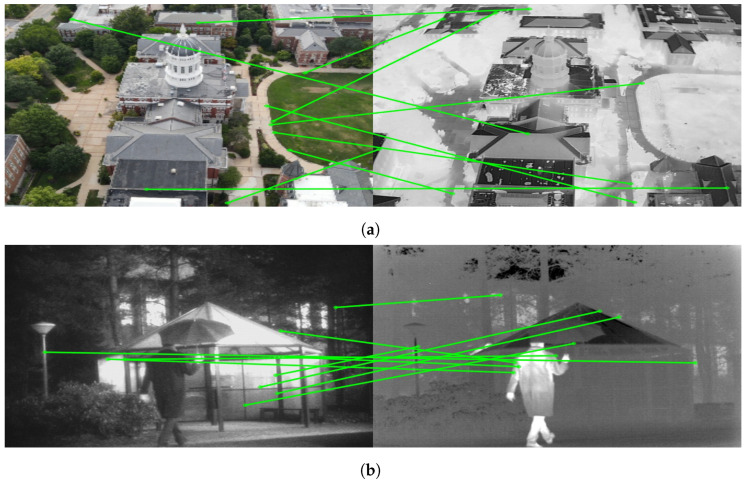

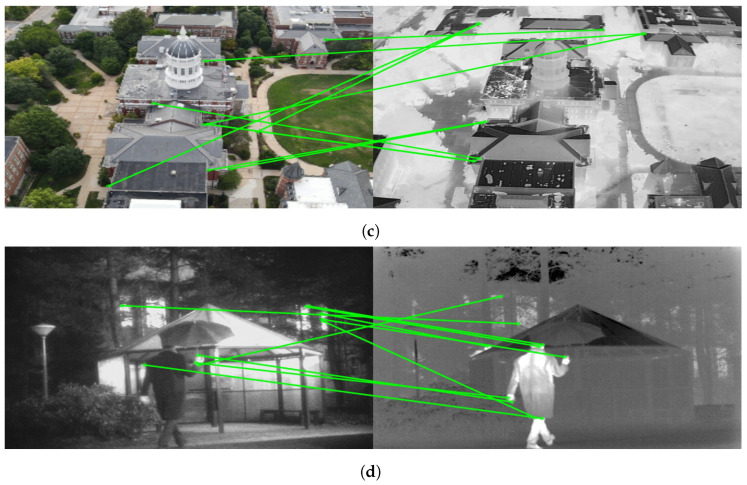
Performance of SIFT (**a**,**b**), ORB (**c**,**d**) feature detection and matching applied to two sample images from JesseHall and Kittler datasets. The algorithm detects individual key points at different locations in each modality but failed to find the correlative matches highlighting the limitations of classical methods applied to multi-modal image matching.

Feature-based image matching methods extract robust multi-scale features from the underlying data and encode them into unique descriptors which remain the same for variations in scale and other real-time operations. For example, SIFT [21] uses difference of Gaussian (DoG)-based image decomposition and extrema selection to identify keypoints in the image and encode them into histogram-based descriptor. Prior to SIFT, classical image processing methods used filtering and PCA-based methods for image correlation which were extremely susceptible to scaling and other operations. The success and robustness of SIFT accelerated the development of feature-based methods for image matching. The work was followed by other algorithms such as ORB [22] and SURF [23]. These feature-based methods were very successful in matching images of the same modality, but they are not suitable for matching between images from different modalities, where the underlying image data vary drastically based on the modality of capture. This cannot be correlated for designing a common encoding vector, and since these methods use handcrafted features for encoding with no leeway for any improvements, they fail to robustly match multi-modal images, as seen in Figure 3.

#### 2.1.3. DNN-Based Image Feature Matching

The methods described above lack the ability to adapt the algorithms with the variations in the quality of input which severely limits the usage of these methods for real-time implementation. Deep neural networks (DNN) have successfully addressed this problem by incorporating a learning component in their design [24,25,26]. The DNN-based methods are trainable for adapting to variety of input scenarios and are capable to produce improved output. Ref. [27] presented CoMIR: Contrastive multi-modal Image Representation for Registration method of multi-modal images. The proposed network reduces the multi-modal registration problem to a mono-modal one which facilitates the use of intensity-based and feature-based registration algorithms. In [28] authors presents a similar work based on image-to-image translation network. They developed a geometry preserving image-to-image translation network which allows comparing the deformed and target image using simple mono-modality metrics. A review of different multi-modal image matching algorithms is concisely presented aong with a comparative analysis [29]. Superpoint is a DNN-based state-of-the-art keypoint detector for image matching trained on generic corner and edge images which successfully generalizes the ability of the network to work with variety of datasets [30]. The detector though trained on visible data only, performed reasonably well on multi-modal data which is explained in results and discussion. Though it provided the best performance among existing methods, the reliability of the algorithm was not ensured as the performance of the algorithm was highly dependent on the quality, texture, and spatial frequency content of the images. The main shortcomings of these previous methods are discussed in the results section along with the proposed solution. Visible and infrared image matching is a much more challenging problem due to variations in sensor parameters, quality of output, data representation, and variation in output with ambient lighting, weather conditions, etc. It is widely used in autonomous driving [31], tracking [32,33], and segmentation [34] applications. Some of the relevant works in this area are given in [35,36,37] for the interested reader.

### 2.2. Multi-Modal Image Fusion

Fusion methods can also be broadly classified into (a) Classical Multi-scale methods [38,39,40,41], (b) Saliency-based methods [42,43] and (c) DNN-based methods [44,45,46]. Classical methods relied on mathematical models or dictionary elements to understand and fuse the multi-modal content. These methods have been effective but are limited in their ability to handle complex feature representations. In [47], the authors presented a Laplacian filtering-based image decomposition into low- and high-frequency bands. The low-frequency bands are processed using sparse representation and the high-frequency bands are processed using guided filtering combined with the weighted sum of eight neighborhood-based modified Laplacian operator. The processed bands are passed through an inverse Laplace processing module to generate a fused image. They also extended their approach for conducting fusion experiments on multi-focus images, achieving satisfactory results in capturing diverse focal points within a single fused output. Ref. [48] proposed an image fusion method based on feature-based decomposition and domain normalization. The decomposition method separates infrared and visible images into common and unique regions and applies domain normalization to the common regions to generate a unified feature space. This space is used to transform infrared features into pseudo-visible domain, ensuring that the fusion is implemented in this common domain, minimizing the impact of modal differences during the fusion process. They also proposed the non-local Gaussian filter to minimize the effects of noise without compromising on fusion quality. Ref. [49] presented an exhaustive template search for image matching and autoencoder for fusion. The experiments were performed on benign image pairs with maximum overlap for registration. For a general scenario, with no information on the range of overlap this method is highly computationally intensive and may not converge in real-time.

Deep learning methods have quickly emerged as an efficient alternative to classical approaches based on their ability to analyze multi-modal data at a much more structural level. This can be seen from the large number of publications in multi-modal image analysis based on DNNs over the past decade. There are several works using deep learning architectures for image fusion. They can be divided into (a) multi-stage pipelines [50,51] and (b) end-to-end pipelines [52,53]. Multi-stage pipelines have more flexibility, but they are highly susceptible to coherence loss which occurs due to independent processing modules. End-to-end systems, on the other hand, directly encode the parameters in a single connected pipeline which preserves the relationships and results in improved fusion performance. In [54], authors compiled several fusion methods published in the past decade and concisely summarize the performances. A detection and tracking system of small UAVs using VIS-IR image fusion is presented in [55]. They proposed two data integration techniques, one at the decision level and the other at the pixel level. At the decision level, the VIS and IR inputs are processed separately and are passed onto an image fusion module for detection and tracking. In decision level processing, the inputs are classified as primary and confirmatory. The primary image is used for detection and tracking with a confirmatory image used for confirming the target; pixel-level processing is performed using FusionGAN [45].

Multi-modal fusion has also been successfully applied in a variety of real-time applications like segmentation for autonomous driver assistance system (ADAS) applications. Research on autonomous systems has increased rapidly over the past decade for both ground-based and aerial applications. These are safety prioritized applications which directly affect human safety and infrastructure, and need to operate continuously all-day in all-weather conditions. Adding infrared cameras to the ADAS sensor suite improves scene perception, resulting in better system performance as described in [56,57,58,59].

### 2.3. Fusion Performance Metrics

Although there is a great deal of published literature on image fusion, the problem is still open for research. The primary reason for this is the lack of a universally applicable metric for analyzing the multi-modal fusion performance. The works published in this domain considered metrics developed for the visible systems for performance evaluation and extended them to evaluate VIS-IR systems. However, the metrics developed for visual systems such as SSIM [60], normalized cross-correlation [61], average gradient [62], Qabf [63], mutual information [64], spatial frequency [65], etc., assume some level of correlation between input images like focus altered images, scaled images, etc. However, applying them to multi-modal image is purely experimental, and therefore the results produced are not universally acceptable resulting in so many application-based publications. In this work, we propose a keypoint-based analysis which natively compares the fused output with the input images. Keypoints hold significant information from individual modalities which can be leveraged to quantify the fused output in a much more data centric way relative to the input images. In [66], the authors present the use of keypoints for clustering in medical images.

## 3. DeepFusion Pipeline

### 3.1. Wavelet Spectral Decomposition (WSD)-Based Multi-Modal Image Matching

Photodetectors for visible sensors are widely established, offering very high resolutions and expansive fields of view (FOV). Current state-of-the-art sensors boast Ultra HD (UHD) resolutions up to 8K pixels, with FOVs ranging from $90^{\circ }$ to $120^{\circ }$. Due to densely packed pixels in visible detectors, variations in FOV minimally affect image quality. However, these sensors are limited by operational range and ambient lighting conditions. In contrast, infrared sensors are highly heat-sensitive, complicating both detector and housing designs. Present day state-of-the-art infrared sensors come with full HD resolution (1K) which is 8 times lower than visible systems. Due to this low resolution, the quality of the infrared image captured is highly sensitive to field of view and the quality of infrared output degrades significantly with small changes in FOV. Therefore, commercially available infrared systems come with smaller FOVs limiting the capture of information from the scene. For the present work, visible images are captured at 6K × 4K resolution and infrared images are captured at 640 × 512. On the other hand, the image quality of visible images degrades exponentially with minor changes in ambient conditions whereas infrared sensors are relatively stable but have poor contrast and texture information. To design a system for day and night operation, the output from both the sensors should be judiciously fused. The first problem to address in multi-modal fusion is mapping the low-resolution infrared equivalent information represented in the high-resolution visible frame. This is a challenging problem because there is no direct way available to automatically pinpoint the subframe in visible image representing infrared information. Image matching techniques have been extensively studied for comparing visible images under various conditions such as focus quality, noise, and environmental factors. These methods typically fall into two categories: (a) data-based methods and (b) deep neural network (DNN)-based methods. However, these approaches often struggle when applied to multi-modal images due to inherent differences in data distribution between sensors.

**Figure 4:**
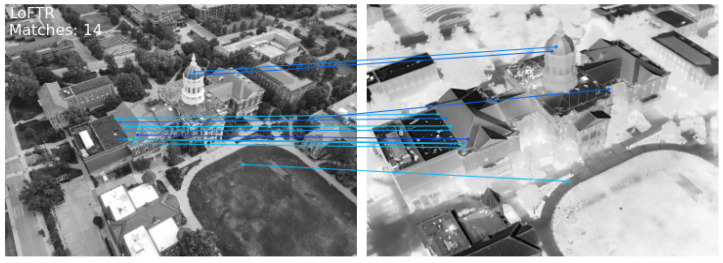
Performance of LoFTR [21] feature matching on a JesseHall image pair. The algorithm detects individual key points at different locations in each modality but fails to match them correctly. It clearly indicates that multi-modal sensors capture the scene information quite differently.

**Figure 5:**
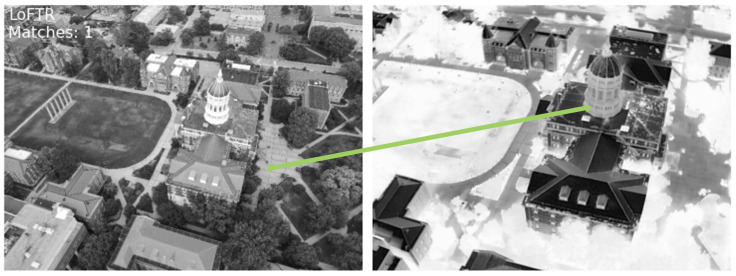
Performance of LoFTR feature matching on another JesseHall image pair. The algorithm failed to detect individual key points in the images.

**Figure 6:**
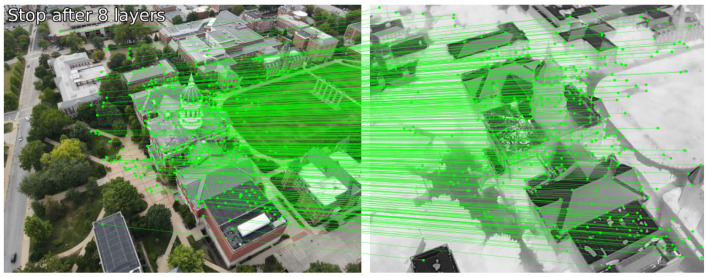
Image matching using LightGlue. The keypoints and matching performance of the network is better than other methods. However, there are still incorrect correlations which result in errors for homography estimation.

**Figure 7:**
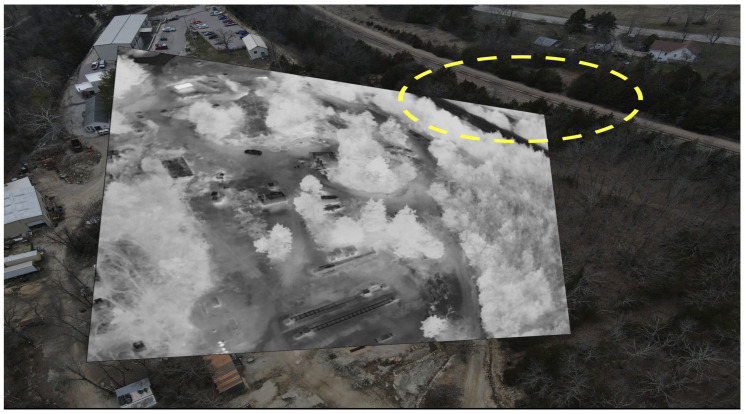
Homography estimation of an IR image in the visible frame using the LightGlue network. The homography matrix generated a skewed mask which can be observed in the highlighted location for sample image from the Rolla dataset. The skewness worsens for the images captured during the turns amplifying the minor positional differences of the visible and infrared sources.

**Figure 8:**
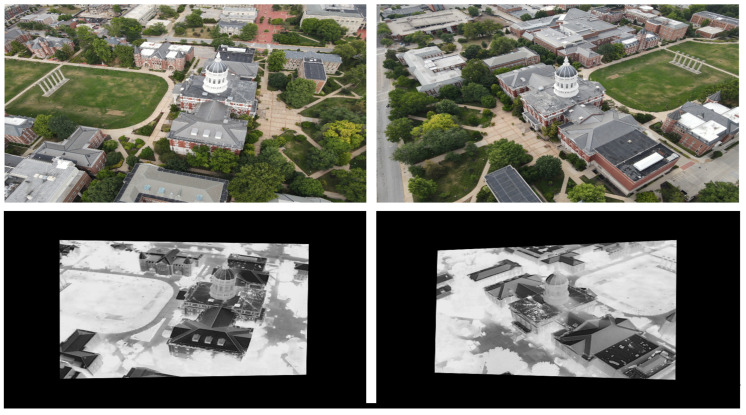
Results of homography estimation using keypoints generated by the Superpoint algorithm matched with LightGlue network for the 2 images from the JesseHall dataset. The mask generated using a homography matrix is skewed across the edges, as seen in the figures, due to the difference in spatial orientation of the VIS-IR sensors which introduces fusion artifacts in the output.

Figure 3 illustrates the output of classical keypoint-based descriptor matching using SIFT [21] and ORB [22] features for a pair of visible-infrared (VIS-IR) images. It can be observed that keypoints in both images are located differently, and even co-located keypoints do not have matching descriptors due to variations in underlying pixel data. Additionally, keypoints at different locations sometimes exhibit similar descriptors. These observations demonstrate the limitations of keypoint-based methods for multi-modal image pairs. To address these challenges, we explored DNN-based solutions, which have emerged as a promising alternative in computer vision. Figure 4 and Figure 5 depict the results obtained using LoFTR [67] for the image pair from the JesseHall dataset. Figure 4 shows good keypoint matches among incorrect matches, but the performance of the network for the image pair captured under different perspective resulted in no matches as shown in Figure 5. Further experimentation involved employing the state-of-the-art LightGlue [68] CNN with the Superpoint [30] detector. This approach showed improvement in image matching quality as shown in Figure 6, but the match quality was not reliable for changes in perspective. Apart from reliability, the homography estimation, crucial for alignment, also produced skewed masks, as depicted in Figure 7 for the MU Rolla dataset. Figure 8 shows homography masks for other image pairs from the JesseHall dataset. It is also observed that minor variations in camera positions exacerbated this issue, highlighting the sensitivity of these methods to spatial orientation. So, while DNN-based methods offer flexibility and potential improvements over classical techniques for multi-modal image matching, challenges remain in achieving robust and accurate results, particularly concerning spatial alignment and descriptor matching across different sensor modalities.

Our experiments conclusively demonstrate that classical keypoint and deep neural network (DNN)-based image matching techniques fall short when applied to multi-modal image processing. This necessitates the need for novel and more sophisticated approaches when dealing with diverse imaging modalities, such as visible light and thermal imagery. The human visual system (HVS) possesses an innate ability to discern similarities between multi-modal images. In contrast, algorithms must laboriously derive these relationships from raw pixel data, presenting a significant challenge in automated image processing. In [13], the authors presented an empirical relationship between visible and thermal images and in [69], the authors propose Siamese-based DNN in which they show that the visible and thermal features vectors correlate in higher dimensions using a tSNE embedding plot. Since HVS is sensitive to high-frequency spatial information, we can map the images visually. However, to automate the process, a real-time algorithm must capture and correlate this spatial spectral information. To extract this spectral information from both the images, we decomposed the images using a wavelet transform with Daubechies wavelets [70]. First-level 2D wavelet decomposition yields four spectral quadrants (low–low (LL), low–high (LH), high–low (HL), and high–high (HH)) defined as,

(2) $$\phi_{j,m,n}\left(x,y\right)=2^{j/2}\phi \left(2^{j}x-m,2^{j}y-n\right)$$

(3) $$\psi_{j,m,n}^{i}\left(x,y\right)=2^{j/2}\psi^{i}\left(2^{j}x-m,2^{p}y-n\right),i\in \left(H,V,D\right)$$

The low-frequency quadrant holds approximation coefficients which are typically utilized in lossy image compression applications (e.g., JPEG2000 [71] standard) and high-frequency information encapsulates crucial edge map information, which is used for precise image matching using Equations (4)–(9).

(4) $$I_{\mathrm{DWT}}^{VIS}=G\left[F\left(I_{VIS}\right)\right]$$

(5) $$F\left(I_{VIS}\right)=\sum_{i}\psi_{\mathrm{DWT}}^{i}\left(I_{VIS}\right),\;\;i\in \left(H,V,D\right)$$

(6) $$I_{\mathrm{DWT}}^{IR}=\left[F\left(I_{IR}\right)\right]$$

(7) $$F\left(I_{IR}\right)=\sum_{i}\psi_{\mathrm{DWT}}^{i}\left(I_{IR}\right),\;\;i\in \left(H,V,D\right)$$

**Figure 9:**
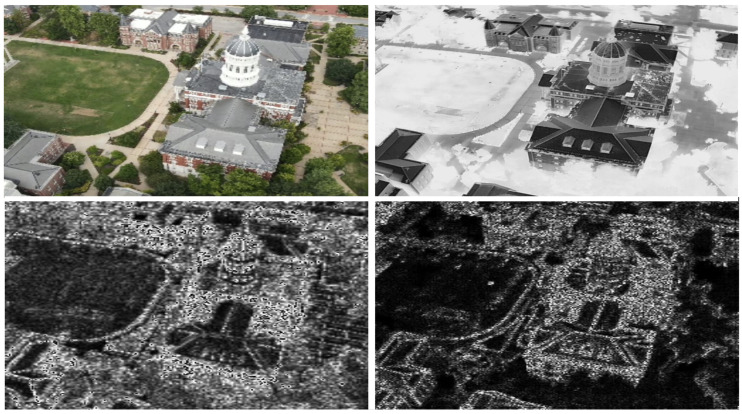
Images show the matched edge maps for VIS-IR image pair from the JesseHall dataset. The images are decomposed using Daubechies wavelet. The information from low-frequency quadrant is discarded and the high-frequency quadrants capturing edge information (H, V, D) are processed to generate a fused edge map. The edge maps are iteratively processed to find the local SSIM maxima to generate a registered image pair as shown in top row.

The function F(·) represents the mixing and scaling of high-frequency quadrants followed by a Gaussian filtering represented by G(·). Gaussian filtering was not applied to the thermal image which uses longer wavelengths and the micro-bolometer sensor has lower resolution for measuring emitted infrared energy. After image filtering, the SSIM index between the processed VIS-IR edge maps is calculated and the process is repeated until the SSIM index reaches a local maxima,

(8) $$s^{*}=\underset{s\in \{VIS\_Crops\}}{\mathrm{argmax}}SSIM\left(I_{VIS}\left(s\right),I_{IR}\right)$$

where argmax of *s* searches over a range of scales based on a set of boundary cropping the visible image to find $s^{*}$ which is the best crop that yields an SSIM local maxima. After finding the best scale, $s^{*}$, optimizing the SSIM measure, the registered visible image patch ($I_{VIS}^{Reg}$) is extracted from the high resolution visible image as,

(9) $$I_{VIS}^{Reg}=T\left(I_{VIS},\;s^{*}\right)$$

where $I_{VIS}^{Reg}$ is the final registered visible input matching thermal information and T the scaling, or in general a homography, transformation to align the VIS and IR images. Figure 9 shows the output of the WSD-processed JesseHall image pair. It can be observed that the both the modalities share a similar edge map structure to the predicted one, which resulted in the registered image pair shown in the top row of Figure 9. The proposed approach leverages advanced wavelet analysis to extract and process the most pertinent spectral information for multi-modal image matching, as explained in Equations (2)–(9) above. By doing so, it aims to overcome the inherent limitations of conventional feature vector- and DNN-based methods, potentially improving multi-modal image matching as shown in the next section.

### 3.2. Image Fusion

Image fusion is a reliable way of improving information by leveraging complementary features from multi-modal sensors. Efficient fusion techniques aim to maximize the utilization of spatio-temporal correlations and complementary information between visible and thermal sensors, resulting in improved images that are beneficial for both human visual perception and computer vision tasks. Deep learning methods offer promising fusion results due to their capability to learn intricate feature representations directly from source images and fuse them efficiently. Deep learning-based fusion methods operate in two primary modes: (a) multi-stage fusion and (b) end-to-end fusion. Multi-stage fusion methods are flexible, allowing for the integration of various preprocessing techniques across different stages. However, they can be computationally intensive and prone to information loss during inter-stage processing. End-to-end fusion methods, on the other hand, are more efficient in preserving information. These methods employ a single deep learning model that takes multiple input images and directly outputs a processed image. They learn the optimal fusion strategy from raw input images without prior knowledge of the fusion process, accommodating thermal fusion for black hot and white-hot images naturally. However, designing effective end-to-end fusion models can be challenging and requires careful consideration of loss functions to achieve optimal performance. Despite the extensive research in this field, several challenges still remain.

Firstly, existing fusion methods are typically designed for normal lighting conditions in both visible and thermal images, neglecting the exponential degradation of image quality in varying ambient conditions. One approach to mitigate this issue is preprocessing images for illumination adjustment before fusion, although this can sometimes amplify artifacts indirectly associated with object characteristics. Secondly, there is no universally accepted metric for comparing the quality of fused images against raw multi-modal inputs. Existing metrics such as Qabf [63], MSE [72], the Chen-Blum metric [73], and the Chen-Varshney metric [74] typically focus on pixel-level correlations.

**Figure 10:**
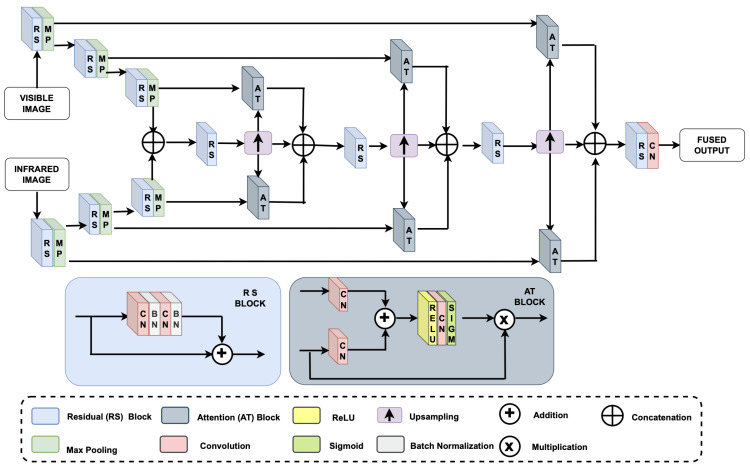
Block diagram of the proposed VIRFusionNet image fusion module. The network is designed as a multi-channel encoder–decoder architecture with attention blocks focusing on extracting dominant features from both the modalities at different levels of decomposition. The encoder blocks are designed with stacked residual and max pooling blocks which are concatenated and processed through the decoder. The network employs skip connection to multi-level attention blocks which focus on extracting relevant information from both the modalities for enhanced fusion. The sub-figures below show the detailed architecture for residual and attention blocks.

In this work, we propose the *VIRFusionNet* network with skip connections and attention modules for image fusion as shown in Figure 10. The model is based on an encoder–decoder architecture with parallel input channels for registered VIS-IR image pairs. Each encoder consists of residual blocks followed by max-pooling layers. Residual blocks help in learning complex features, while max-pooling layers reduce spatial dimensions for computational efficiency and capturing essential features at different scales. The encoded feature vectors from both the inputs are concatenated to leverage complementary information from both the images, facilitating better feature integration and representation. The concatenation also increases the feature space, enabling more comprehensive learning. The decoder module uses up-sampling layers to increase the spatial resolution to compute attention maps for the network to focus on relevant features from both modalities, enhancing the final fused image quality. The network is trained with equally weighted hybrid loss function optimized for MSE and SSIM parameters. Additionally, we introduce keypoint-based analysis for assessing the image quality, aiming to quantify the preservation of original image content in the fused output. This metric addresses the need for evaluating fusion quality beyond traditional pixel-level comparisons, offering insights into how well the fused image retains meaningful raw data. By tackling these challenges, we aim to improve image fusion, enhancing its applicability in various domains such as surveillance, medical imaging, and remote sensing.

### 3.3. Keypoint-Based Quality Analysis

SIFT (scale-invariant feature transform) introduced keypoints as a robust method for encoding image information. Its descriptor captures local image details around keypoints, preserving this information even when the image is scaled or rotated. SIFT has been successfully applied in various applications such as image stitching [75,76], 3D reconstruction [77], and structure from motion [78]. While it may not directly facilitate image matching for multi-modal images, keypoints can serve as an important tool for quantifying the quality of fused images.

(10) $$[K_{Vis},D_{Vis}]=K_{P}\left(I_{vis}\right)$$

(11) $$[K_{IR},D_{IR}]=K_{P}\left(I_{IR}\right)$$

(12) $$[K_{Fused},D_{Fused}]=K_{P}\left(I_{Fused}\right)$$

where $K_{P}\left(I_{vis}\right)$ is a keypoint operator that returns the location of the detected feature point, $K_{Vis}$, and its associated description vector $D_{Vis}$. Similarly, $K_{P}\left(I_{IR}\right)$ and $K_{P}\left(I_{Fused}\right)$ are the keypoint operators applied to the IR and fused images. We use primarily the feature point locations in the matching and evaluation process. The objective of image fusion is to enhance information using multiple sensor modalities while preserving the unique details from each modality without compromising raw pixel data. Keypoints provide an efficient means to assess the quality of information in the fused output. They can help in evaluating how well the fused image retains native exclusive content from both visible and thermal modalities. For the present case, we have two ground truth elements (VIS, IR images) and the fused output. It is observed from images above that traditional keypoint matching fails for multi-modal images due to inherent difference in device physics of data capture. However, the key point locations can serve as a baseline reference to assess the fusion quality. To address this, we define *F1 score* [66] based on the precision and recall metrics using keypoints as defined in Equations (16)–(18) to assess the quality of the fused output.

(13) $$TP=\forall_{\left(K_{Vis},K_{IR}\right)}(∥K_{Fused}-K_{Vis}{∥}_{2},∥K_{Fused}-K_{IR}{∥}_{2})<n$$

(14) $$FN=\forall_{\left(K_{Vis},K_{IR}\right)}(∥K_{Fused}-K_{Vis}{∥}_{2},∥K_{Fused}-K_{IR}{∥}_{2})>n$$

(15) $$FP=\forall_{\left(K_{Fused}\right)}\;NOT\;detected\;in\;\left(K_{Vis},K_{IR}\right)$$

(16) $$Precision=\frac{TP}{TP+FP}$$

(17) $$Recall=\frac{TP}{TP+FN}$$

(18) $$F1\;Score=\frac{TP}{TP+\frac{FP+FN}{2}}=\frac{2*Precision*Recall}{Precision+Recall}$$

**Figure 11:**
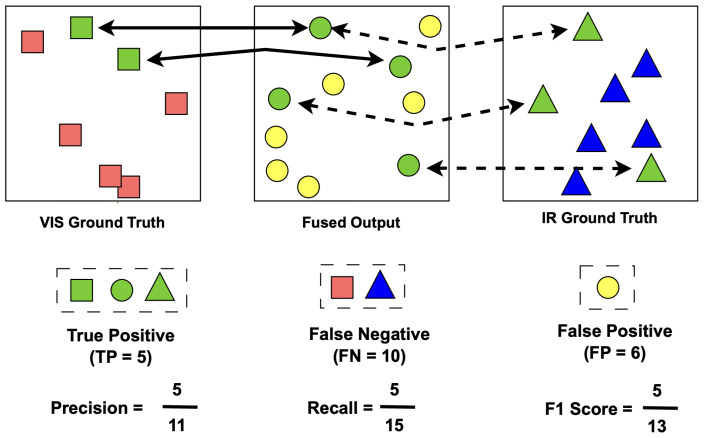
Cross-channel feature detector performance evaluation. Top row illustrates the definition for matched feature points that are true positives (TP, green in VIS or IR), false positives (FP, yellow in Fused) and false negatives (FN, red in VIS and blue in IR). Reproducibility of keypoints between the Fused image and those detected in the original VIS and IR images, is quantified using precision (Prec), recall (Re), and F1-score (F1) to evaluate fusion performance (example in bottom row).

We define *TP* true positives, *FP* false positives, and *FN* false negatives for VIS, IR and Fused images. Figure 11 shows the method of evaluating these metrics based on keypoints as defined in Equations (13) to (15). The True positives are the keypoint locations geometrically matched with a tolerance window *n* between (VIS-GT, Fused) and (IR-GT, Fused) images. False Positives are the keypoints not present in the ground truths but are detected in the fused output. These can also be termed as fusion artifacts. False Negatives are the original keypoints present in ground truths which are not detected in the fused output. The fusion method which can preserve the keypoints in fused output will result in high *TP* and low (*FP*, *FN*) values which are used to quantify the performance of image fusion. We then define the *F1 score* [66] based on Precision and Recall metrics using keypoints as in Equations (16) to (18), to assess the overall quality of the fused output. The proposed DeepFusion pipeline successfully captures the maximum native information from ground truth, as explained in later sections.

For the present work, we considered the SIFT [21] detector. Descriptors $D_{Vis}$ and $D_{IR}$ are not used here because of the discrepancy in the underlying data, which will create a wrong association for the matcher. We compared the fusion performance of open-source networks with proposed network using standard single-mode metrics alongside the proposed keypoint-based analysis. Single-mode metrics typically evaluate fused images based on traditional measures like image sharpness, contrast, and noise levels, which are crucial but may not preserve the original information from each modality effectively. By introducing keypoint-based evaluation, we aim to provide a more nuanced assessment of fusion quality. This approach considers how well the fused image maintains distinct features identified by keypoints originally present in the individual modalities. It allows for a deeper understanding of whether the fusion process successfully integrates and enhances information from both visible and infrared sensors. While traditional metrics provide valuable insights into overall image quality, keypoint-based evaluation offers a focused analysis on the preservation of unique details from input modalities with minimal data corruption. This dual assessment (traditional and keypoint-based) strategy helps in comprehensively evaluating the performance of image fusion techniques across different modalities and scenarios.

## 4. Results and Discussion

In this section, we present the details of the datasets considered for experimentation and the implementation aspects. We present quantitative and qualitative comparisons of the proposed technique with the state-of-art methods published over the last decade for contextual relevance.

### 4.1. Dataset and Implementation Details

#### 4.1.1. Dataset

Our primary focus is the fusion of VIS-IR images captured from an aerial platform for wide area motion imagery (WAMI) [79,80] applications such as comprehensive structure for motion (SFM) [81,82], geo-registration [83] and WAMI tracking [84]. The datasets are captured with a DJI Mavic 2 Enterprise drone following a predefined elliptical trajectory at a height of 120 m carrying an optical payload of visible and thermal sensors. Several datasets were captured including buildings, fields, construction sites, etc. To compare the performance of the proposed pipeline with published methods we also tested our algorithm on benchmark datasets. In VIFB [54], authors concisely summarized the performance of published fusion methods on *21* image pairs covering different kinds of targets under various lighting conditions. Kittler et. al [85] published another dataset which also has *21* image pairs with unique target locations captured under severe occlusions and lighting conditions. Other open-source datasets considered include RGBT234 [86], RoadScene [87], OSU [88] and the latest CART [89] aerial dataset published by CalTech in 2024.

#### 4.1.2. Implementation Details

The proposed DeepFusion pipeline is implemented in PyTorch version 1.10.1+cu102 and Keras version 2.6.0. The network was trained on NVIDIA Quadro RTX 8000 GPU using the Adam optimizer [90] with an initial learning rate of $10^{-3}$. The network is designed in encoder–decoder format to make it a single flow end-to-end pipeline with skip connections to retain high-resolution features. We also incorporated attention blocks in the decoder architecture for the network to focus on objects in scene for efficient fusion. Encoder channels are designed separately for visible and infrared inputs. The output of both channels are concatenated and forwarded to a single decoder module with attention blocks to generate fused output. The batch size of the experiments was set to 16 after several heuristic tests, and the number of epochs to 50.

### 4.2. Experiments

#### Image Registration

Automating the image registration process for visible-infrared (VIS-IR) image pairs is a complex task due to the differences in focal lengths, fields of view (FOVs), and resolutions between visible and thermal sensors. Traditional fusion methods often assume availability of manually registered image pairs, which can lead to errors and incorrect matching of images. Therefore, automating this registration process is essential for developing real-time multi-modal processing pipelines. However, there is no literature published addressing the automation of image registration specifically tailored for VIS-IR image pairs. To the best of our knowledge, this is the first work in this direction. Key challenges in automating image registration for VIS-IR pairs are (a) sensor characteristics such as focal length, FOV, and resolution; (b) adaptability to varying lighting conditions and environmental factors; and (c) ensuring computational efficiency suitable for real-time applications.

The challenges faced in applying classical keypoint descriptor matching algorithms like SIFT [21] and ORB [22] to cross-modal VIS-IR image pairs highlight significant differences in data structure and characteristics between visible and infrared images. Figure 3 illustrate the limitations of these methods, where keypoints and their descriptors from visible and infrared images fail to match due to inherent disparities in data representation. The mismatched keypoints and descriptors, indicated by slanted lines in the figures, demonstrate the ineffectiveness of traditional approaches in handling multi-modal image matching tasks.

**Figure 12:**
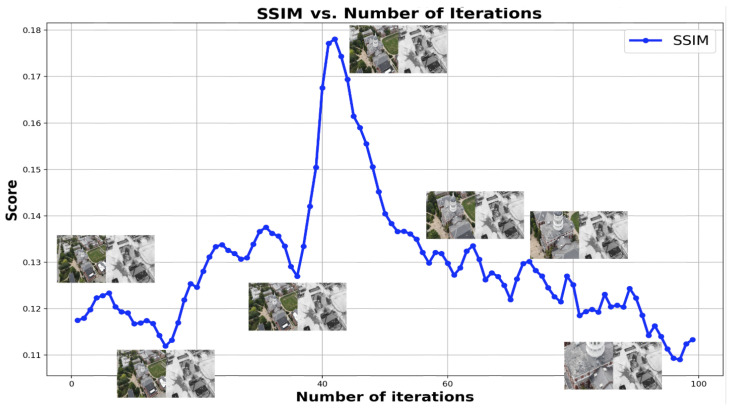
Plot shows the wavelet spectral decomposition (WSD)-based recursive image matching performance mapped with respect to SSIM index. It can be observed that the image pair at the maxima is registered perfectly without any skew as shown. Each image subplot shows the iteratively stripped visible image concatenated with infrared template for comparison.

**Figure 13:**
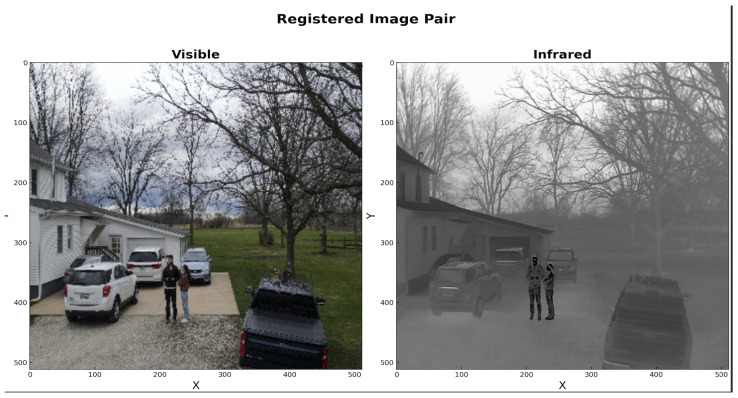
Image matching output using wavelet spectral decomposition and pyramid search method for a sample image pair from TKHouse dataset. The proposed wavelet-based image matching pipeline successfully extracts the matched visible information from high-resolution input.

While DNN-based keypoint generators and matchers offer advancements over classical methods, the challenges of homography-based approaches persist in aerial applications due to the inherent variability in sensor positioning and orientation. To tackle this challenge, we utilized the fact that both sensors capture the same scene albeit with differing camera parameters. Hence, there should be a significant correlation between the image pairs, noticeable even to the human visual system (HVS). The primary task at hand is to accurately estimate the overlapping area between these image pairs, despite lacking specific camera metadata. To correlate the data, we generated edge maps for both the images using DWT-based spectral decomposition. From the decomposed image, the low-frequency content is ignored and the other spectral bands representing high-frequency edges are consolidated to form a search template. The edge maps from visible channel are Gaussian blurred for smoothing the internal edge information in the object data for improved matching performance. Infrared image was also decomposed, and the edge maps are generated for image matching. Working with decomposed data also helped reduce the number of computations by four-fold. We tried a sequential template search method based on spectral decomposition starting from the top-left corner of the visible frame with varying width and height to identify the resolution of mapped infrared content in the visible frame. This brute force method is exponentially time-consuming and cannot be implemented in real-time with no information on the approximate size of the template. Since we know that the sensors are co-located, we modified the spectral decomposition algorithm to perform a crop and scaled strip search for identifying the high-resolution visible subframe representing infrared information. The visible image is stripped from all sides by a predefined width and resized to pass through image matching module based on SSIM [60] index. The process is repeated sequentially and in every iteration SSIM [60] index was calculated to quantify the structural similarity between the infrared template and visible image. This method drastically reduced the computational load by reducing the number of search iterations. For the present case, the method converged within 80 iterations. To further reduce convergence time, we implemented adaptive strip search across the image, however without any information on the template size, this method may overshoot or undershoot the optimum value which may result in incorrect mapping. The method is successfully tested on multiple datasets captured under varying lighting conditions and the results converged in minimal time and error. Figure 12 shows the search template across the dataset plotted over SSIM [60] index. Figure 13 shows an example of image registration for the TKHouse Dataset. After successfully automating the registration process, the image pair is pushed towards the fusion module.

**Figure 14:**
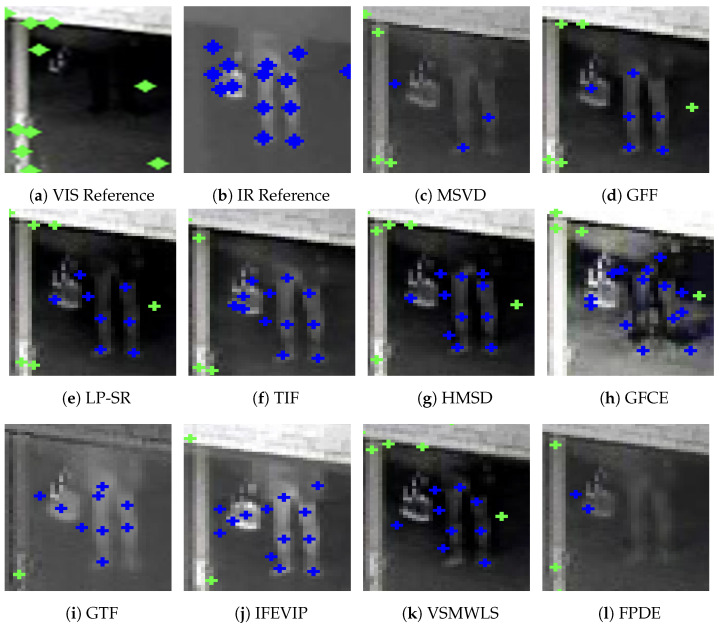

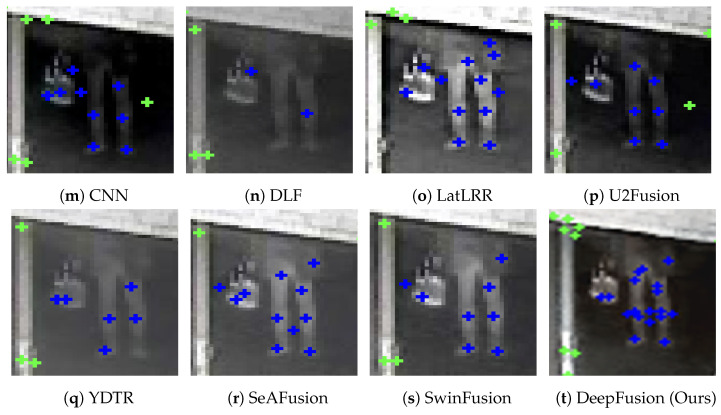
SIFT-Keypoint analysis for VIFB *kettle* image pair for state-of-the-art methods (in chronological order) [85,91,92,93,94,95,96,97,98,99,100,101,102,103,104,105,106] and DeepFusion pipeline. Reference images show the cropped analysis patch (original) for VIS-IR image pair. The rest of the images show the analysis patches of fused output from various state-of-the-art pipelines cropped to show the exclusive keypoints retained from infrared image along with the other detected keypoints. Matched keypoints between fused, visible and infrared images are shown as (plus symbols) Green and the infrared keypoints retained in fused output are shown in Blue.

**Figure 15:**
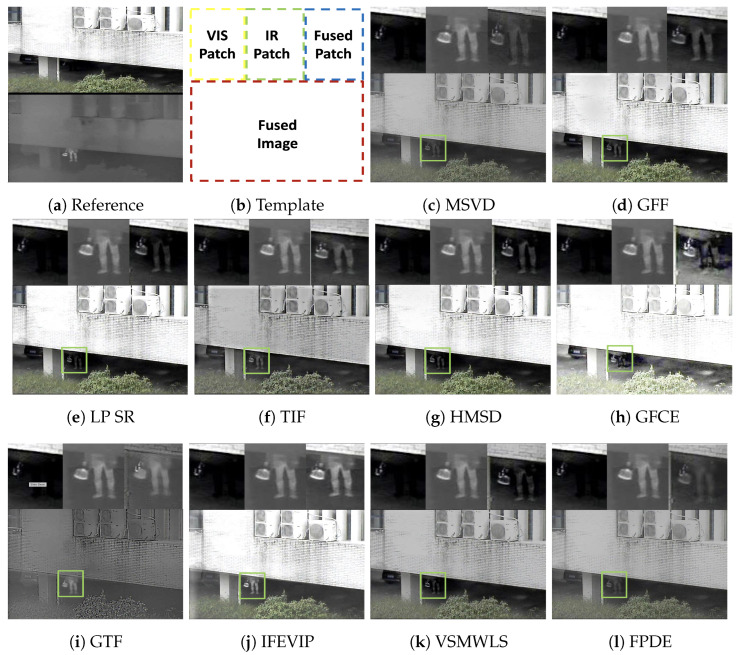

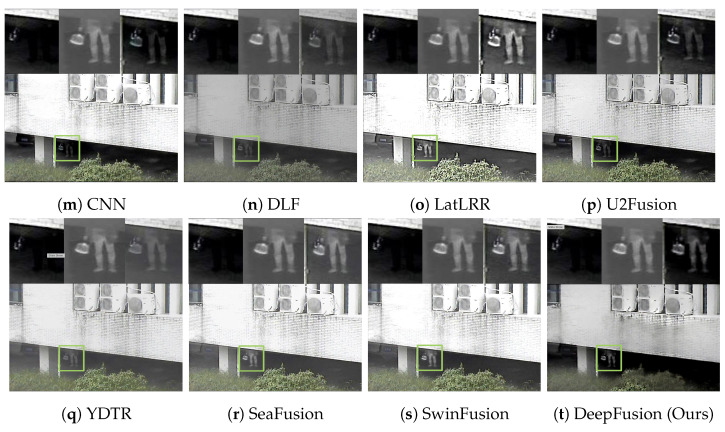
Qualitative performance of state-of-the-art image fusion methods compared to our proposed DeepFusion is shown using the *Kettle* image pair from VIFB dataset with three zoomed inset views, corresponding to the fused image patch shown as a green box [85,91,92,93,94,95,96,97,98,99,100,101,102,103,104,105,106]. (**a**) shows the reference VIS (Top)–IR (Bottom) pair stacked vertically for reference. (**b**) shows the template layout in the rest of the subimages. The three column image patches at the top represent information from visible (VIS), infrared (IR) and fused (Fused) images. The bottom part shows the complete Fused output for each fusion method. The DeepFusion pipeline output in (**t**) better retains saliency and contrast compared to the other methods due to the attention module. Image better viewed in color.

The registered image pairs are forwarded to the VIRFusionNet module, which utilizes an encoder–decoder architecture enhanced with skip connections and attention blocks, as depicted in Figure 10. In this design, attention blocks within the decoder part of the network facilitate the integration of features from various levels of the encoder, each processing different modalities. This strategic integration enables the network to fuse information from both modalities at multiple scales, enhancing the overall integration of multi-modal information in the final output. Figure 14 provides a zoomed-in view of the person in the basement for input VIS-IR images and the fused output to evaluate the fusion performance based on keypoint information. Keypoints serve as indicators of the preservation of original data in the fused image. Observing the figure, our proposed method preserves a significant number of keypoints in the fused image, indicating minimal corruption of original data and effective integration of mutually exclusive information from the input image pair. Figure 15 illustrates a comparison between state-of-the-art DNN-based image fusion models and our proposed DeepFusion approach using the kettle image pair from the Kittler [85] dataset. It can be observed that the visible image misses detecting a person in the basement, a detail captured successfully by the infrared data. This exclusive content image exemplifies the effectiveness of the fusion process in comprehensively integrating multi-modal information. The DeepFusion output effectively retains and combines information from both modalities, as shown in Figure 15.

### 4.3. Performance Analysis

Most of the published methods analyze the fusion performance of multi-modal data based on metrics designed for single-modal systems. However, conventional metrics often overlook the preservation of original information from input images in the fused output. Ideal image fusion should effectively combine complementary information from both modalities while preserving the integrity of the original data as much as possible. To address this limitation, we propose a keypoint-based analysis defined in Equations (13)–(15) to assess the retention of mutually exclusive information from both the input ground truth images in the fused output. Keypoints are particularly suitable for this analysis because they reflect the distribution of original data. However, for completeness of analysis and discussion, we have compared our method with the state-of-the-art networks using the standard visible image matching metrics such as average gradient [62], cross-entropy [107], entropy [108], edge information [109], the Chen–Blum metric [73], the Chen–Varshney metric [74], Qabf [63], mutual information [64], RMSE [72], PSNR [110], SSIM [60] and spatial frequency [65].

**Figure 16:**
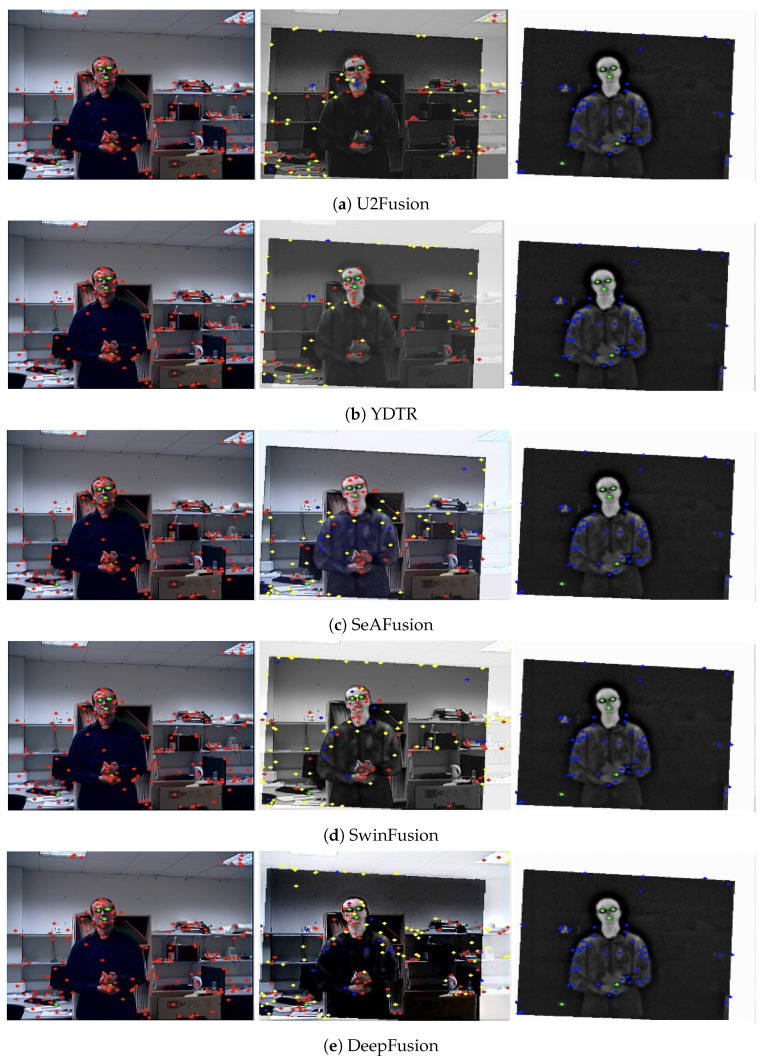
Visualizing feature detections for *LabMan* image pair from Kittler dataset. The keypoints are color-coded based on the modality and location. Green color represents common keypoints in visible, fused output, and infrared images. Red keypoints in first column represent exclusive keypoints present in visible image that are captured in fused image, Yellow keypoints in second column represent false positive keypoints generated in fused output and Blue keypoints in the third column represent exclusive keypoints in infrared image that are also retained in fused output. DeepFusion captures the exclusive keypoints effectively, as seen in the figure, indicating the efficiency of data preservation in the fused output. Image best viewed in color.

**Figure 17:**
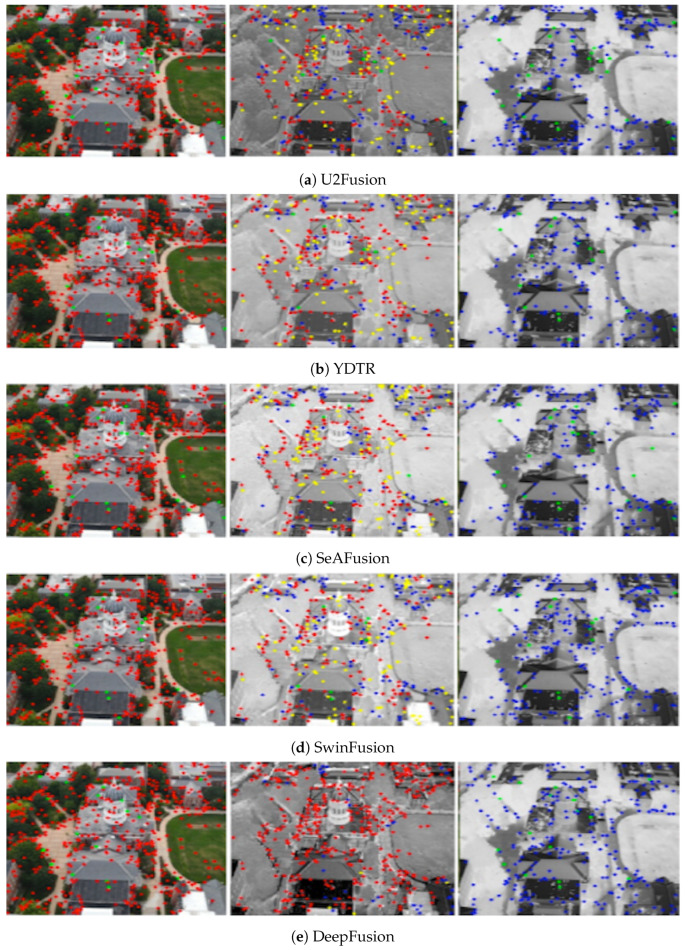
Visualization shows the keypoint plots for another image pair from JesseHall dataset. The keypoints are color coded with Green color representing common keypoints in visible, fused output and infrared images. Red keypoints in first column represent exclusive keypoints present in visible image that are captured in fused image, Yellow keypoints in second column are false positive keypoints generated in the fused output, and Blue keypoints in third column represent exclusive keypoints in infrared image that are also retained in fused output. The DeepFusion pipeline captures the exclusive keypoints effectively as seen from the figure indicating the efficiency of data preservation in the fused output. Image best viewed in color.

In Figure 16 and Figure 17, we compare various fusion techniques based on their ability to retain keypoints in the fused output. It is evident from the figure that the existing methods disrupt the original data distribution during fusion, resulting in a reduction in keypoints and the addition of new keypoints that are not present in the input image pairs. In contrast, our proposed method preserves and even enhances keypoints, as demonstrated in Figure 14 and reduces outliers as shown in Figure 17e. The common keypoints represented as green in fused images of Figure 17a–d are masked by original keypoints from ground truths (red, blue) for DeepFusion indicating improved fusion performance retaining maximum keypoints from VIS and IR ground truth images. As mentioned earlier while emphasizing keypoint analysis, we also evaluated our method using standard metrics for comprehensive comparison and validation. This approach provides a more nuanced evaluation of image fusion techniques, focusing on the fidelity of retained information crucial for applications requiring accurate preservation of original data characteristics across modalities.

Table 1 shows the performance of DeepFusion on some of the open source and custom datasets. It can be observed that no single dataset achieves the best performance across all metrics. Table 2 shows the performance comparison of state-of-the-art fusion methods simulated on the Kittler [85] dataset. Every method has unique strengths, which is evident in the variation in top performance metrics across the models. Red color signifies the top performance and Blue shows the second-best performance. For example, the SwinFusion [106] method has the best PSNR performance and SeAFusion [105] method has the best entropy.

This further highlights the need for a universal metric that can quantify the quality of image fusion. In this work, we propose keypoint-based preliminary analysis, which is the first step in this direction. Table 3 shows the performance of proposed keypoint metric based on F1 score on the VIFB dataset for the proposed and state-of-the-art networks. The tolerance for matching is fixed at 10 pixels. The keypoint vectors are processed using ‘non-max suppression’ to avoid nearby keypoints dominating the metric, resulting in localized evaluation. In Table 3, it can be observed that the performance of DeepFusion is better than the state-of-the-art methods. We evaluated the metric for multiple datasets and the results obtained are reliably consistent. Since keypoints directly relate the original data with the fused output independent of other parameters, they form the ideal metric for comparing the quality of fusion.

## 5. Conclusions

In this work, we addressed the problem of designing a real-time pipeline for multi-modal image fusion for unregistered image pairs. We designed an end-to-end pipeline that takes in multi-modal image pairs and outputs a fused image combining information from both the modalities judiciously to increase the entropy and retain original data in the fused output. To the best of our knowledge, this is the first work in this direction proposing a complete solution. We propose a novel wavelet spectral decomposition-based recursive strip and scale search algorithm to register the image pairs and scale them to a common resolution palette. The registered image pairs are then passed onto the VIRFusionNet module with attention blocks for image fusion. We also propose a novel keypoint metric to quantify the original content retained in the fused image to identify the extent of data corruption. The keypoint metric forms an ideal baseline for comparing various proposed fusion architectures. This is the first metric designed exclusively aiming at multi-modal data, and it reliably outputs consistent performance across multiple datasets and pipeline for efficient fusion analysis. We are currently working towards designing a much more comprehensive network which will try to retain the maximum original information from the raw images in the fused output while improving the information content in the processed output which will successfully support in designing various real-time computer vision and UAV-based applications.

## Figures and Tables

**Table 1:** Performance of state-of-the-art deep architectures for visible and infrared fusion compared to DeepFusion for the Kittler dataset using image restoration quality measures. ‘↑’, ‘↓’ indicate the gradient direction for better performance and ‘→’ shows the DNNs tested for comparison. Red color indicates best performance achieved by the network for the metric.

Network (→) Metric	Deep Fusion (Ours)	SeA Fusion	Swin Fusion	U2 Fusion	YDTR
PSNR ↑	62.2142	63.6008	66.1444	62.0929	63.5828
RMSE ↓	0.2642	0.2213	0.226	0.1754	0.1866
SSIM ↑	1.3252	1.0737	1.1487	1.1992	1.2241
Variance ↑	40.8556	40.7661	36.3481	21.8177	22.9194
Mutual Information ↑	0.7628	1.6846	1.8256	1.0549	1.2813
Entropy ↑	7.1268	6.9883	6.7096	6.1869	6.178
Cross Entropy ↓	1.964	1.6284	1.3206	1.3869	1.053
Average Gradient ↑	6.1181	5.644	4.752	4.0065	3.0561
Edge Intensity ↑	57.3388	55.051	46.7207	38.9731	30.2318
$Q_{abf}$ ↑	0.1414	0.3728	0.3896	0.3121	0.2837
Chen–Blum ↑	0.3485	0.4348	0.4416	0.4524	0.4376
Chen–Varshney ↓	1685.9	748	767.1	839.2	802.7
Spatial Frequency ↑	17.9543	14.826	12.3796	10.1304	7.8594
Average Rank (Top-1) ↑	**6/13**	1/13	3/13	2/13	1/13

**Table 2:** Performance of DeepFusion pipeline on open source and custom datasets. ‘↑’, ‘↓’ indicate the gradient direction for better performance and ‘→’ shows the datasets tested for comparative analysis. The variation in performance between datasets indicates the lack of uniform metric for robust comparison and analysis. Red indicates top performance and Blue represents the next best performance.

Dataset (→) Metric	Jesse Hall (Gaussian, σ=9)	Kittler	VIFB	Rolla	Cart
PSNR ↑	61.7586	62.2142	62.1937	62.5957	62.3601
RMSE ↓	0.2668	0.2642	0.3102	0.2812	0.2669
SSIM ↑	1.1752	1.3252	1.2034	0.6392	0.9818
Variance ↑	30.0103	40.2556	54.4031	42.3442	48.2999
Mutual Information ↑	0.7451	0.7628	0.882	0.4608	0.7971
Entropy ↑	6.8014	6.7268	6.5993	6.6426	6.6503
Cross Entropy ↓	1.9155	1.964	0.7745	0.5571	0.0302
Average Gradient ↑	6.9558	6.1181	6.7735	9.9162	7.7682
Edge Intensity ↑	68.2244	57.3388	67.9655	92.069	71.5114
$Q_{abf}$ ↑	0.1337	0.1414	0.102	0.1392	0.1446
Chen–Blum ↑	0.3427	0.3485	0.295	0.3935	0.3712
Chen–Varshney ↓	1778.6	1685.9	2989.1025	1235.8	2134.1
Spatial Frequency ↑	19.5658	17.9543	25.1461	28.083	22.7736

**Table 3:** Keypoint-based precision (Prec.), recall (Re.) and F1 score (F1) values for various fusion methods on VIFB dataset. The value are derived considering top 100 keypoints in each image with a non-max suppression (NMS) and tolerance window of 10 pixels around the keypoints. The best values of F1 score are shown in Red achieved by the state-of-the-art pipelines. The proposed DeepFusion pipelines achieves the best average output across the dataset with images captured under varied illumination and background conditions.

VIFB	DeepFusion (Ours)			SeAFusion			SwinFusion			U2Fusion		
Image Pair	Prec.	Re.	F1	Prec.	Re.	F1	Prec.	Re.	F1	Prec.	Re.	F1
Car Light	0.440	0.535	0.483	0.522	0.574	0.547	0.600	0.529	0.562	0.611	0.534	0.571
Car Shadow	0.656	0.623	0.639	0.681	0.695	0.688	0.714	0.567	0.632	0.716	0.690	0.703
Car White	0.573	0.530	0.551	0.548	0.454	0.497	0.528	0.546	0.537	0.625	0.380	0.472
Elec Bike	0.410	0.479	0.442	0.402	0.604	0.483	0.396	0.656	0.494	0.402	0.792	0.534
Fight	0.782	0.751	0.766	0.716	0.744	0.730	0.812	0.666	0.732	0.627	0.714	0.668
Kettle	0.359	0.435	0.393	0.438	0.584	0.501	0.447	0.547	0.492	0.365	0.435	0.397
Lab Man	0.500	0.536	0.517	0.500	0.579	0.537	0.529	0.583	0.555	0.518	0.588	0.551
Man	0.392	0.793	0.526	0.407	0.612	0.489	0.444	0.607	0.512	0.436	0.707	0.539
Man Call	0.421	0.708	0.528	0.370	0.554	0.444	0.371	0.629	0.465	0.443	0.507	0.473
Man Car	0.574	0.522	0.547	0.593	0.528	0.559	0.621	0.602	0.611	0.622	0.510	0.561
Man Light	0.750	0.631	0.685	0.671	0.622	0.646	0.671	0.581	0.623	0.589	0.649	0.617
Man Walking	0.457	0.540	0.495	0.410	0.530	0.462	0.455	0.630	0.528	0.478	0.560	0.516
Man with Bag	0.372	0.657	0.474	0.433	0.562	0.489	0.359	0.428	0.391	0.413	0.482	0.445
Night Car	0.466	0.583	0.518	0.387	0.580	0.465	0.408	0.559	0.471	0.543	0.530	0.536
People Shadow	0.736	0.670	0.694	0.735	0.610	0.667	0.661	0.598	0.628	0.607	0.662	0.635
Running	0.681	0.565	0.618	0.721	0.540	0.618	0.723	0.584	0.646	0.654	0.493	0.563
Snow	0.625	0.532	0.573	0.520	0.530	0.525	0.542	0.635	0.585	0.426	0.425	0.425
Tricycle	0.659	0.550	0.600	0.610	0.622	0.616	0.635	0.581	0.607	0.625	0.673	0.648
Walking	0.690	0.645	0.667	0.588	0.561	0.574	0.551	0.609	0.579	0.630	0.640	0.635
Walking2	0.705	0.598	0.647	0.750	0.616	0.676	0.674	0.649	0.661	0.602	0.639	0.620
Walking Night	0.500	0.590	0.541	0.625	0.644	0.634	0.563	0.658	0.607	0.711	0.479	0.572
**Average**	0.559	0.594	0.576	0.554	0.588	0.564	0.557	0.592	0.572	0.554	0.575	0.561

## Data Availability

Data are contained within the article.
